# Supplementary material for: Hybridized Zoonotic Schistosoma Infections Result in Hybridized Morbidity Profiles: A Clinical Morbidity Study amongst Co-Infected Human Populations of Senegal
Source: Microorganisms. 2021 Aug 20;9(8):1776. doi: 10.3390/microorganisms9081776 (PMC8401530; doi:10.3390/microorganisms9081776)
Supplement: Supplementary file 1 [file microorganisms-09-01776-s001.zip › microorganisms-1292306-supplementary.pdf]

Supplementary Materials

Table S1. Hemoglobin levels (g/l) to diagnose anemia as recommended by the World Health Organization (WHO) [1].

Age	Sex	No anemia	Anemia		
			Mild	Moderate	Severe
5-11 years of age	Both	115 or higher	110-114	80-109	Lower than 80
12-14 years of age	Both	120 or higher	110-119	80-109	Lower than 80
15 years of age and above	Females	120 or higher	110-119	80-109	Lower than 80
	Males	130 or higher	110-129	80-109	Lower than 80

Figure S1. Standard image patterns to score the picture of the liver and diagnose periportal fibrosis as recommended by the standardized WHO Niamey protocol [2,3].

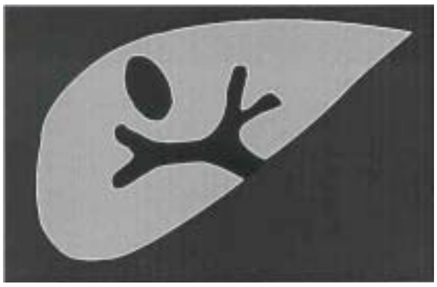

Image pattern A (normal)

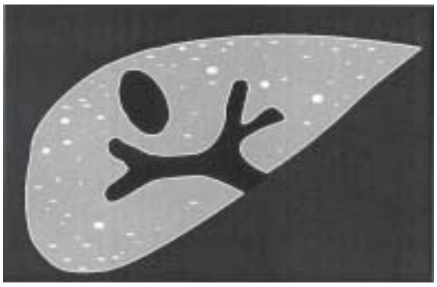

Image pattern B
(peripheral portal branch echogenicity)

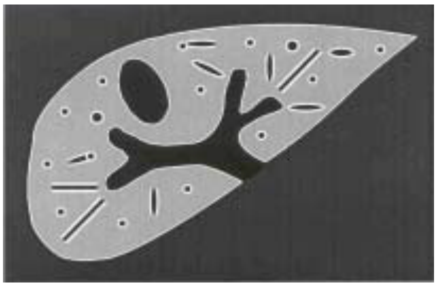

Image pattern C
(peripheral periportal thickening)

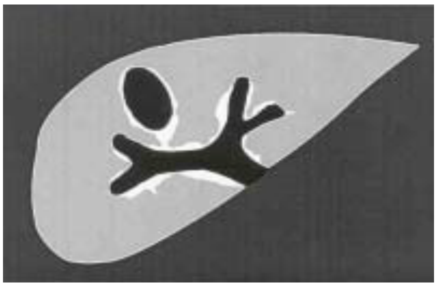

Image pattern D
(central periportal thickening)

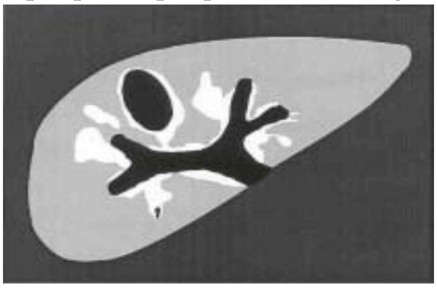

Image pattern E
(central periportal thickening with echogenic patches expanding into the parenchyma)

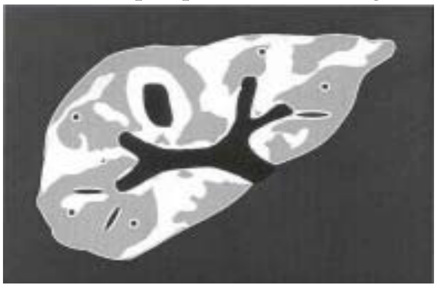

Image pattern F
(very advanced central and peripheral periportal thickening)

Additional Text S1. Cumulative link logistic regression models using R package 'ordinal'.

The R package 'ordinal' was used to fit cumulative link logistic regression models [4], to estimate the effects of urogenital and intestinal infection intensities and their interaction on each morbidity indicator before treatment, adjusting for sex, age and study site (Barkedji or Richard Toll). For children, we accounted for potential clustering of morbidity indicators by defining school as a random effect. We used the logit link, with cumulative logits defined as [5]:

$$\text{logit}[P(Y_i \leq j|x_i)] = \log\frac{P(Y_i \leq j|x_i)}{1 - P(Y_i \leq j|x_i)} = \theta_j - x_i\beta - u_i \quad j = 1, \dots, J-1 \qquad \text{(Equation S1)}$$

where Y_i is a random variable that can fall in $j = 1, \dots, J$ categories (for example, j = +, ++ or +++ for hematuria), θ_j represent the intercepts for each category, x_i is a vector of fixed effects covariates for the ith individual and β is the corresponding set of regression coefficients. Parameter u is the random effects term for school, with $u_i \sim \mathcal{N}(0, {\sigma_u}^2)$. The cumulative logits are defined for all but the last category since for $j = J$ the probability $P(Y_i \leq j) = 1$ (Y_i is necessarily smaller or equal than J).

The regression parameters can be expressed in terms of an odds ratio (OR). For a single dichotomous predictor variable (i.e., $X = 0$ or 1)

$$\frac{P(Y \leq j|X = 1)}{1 - P(Y \leq j|X = 1)} = e^{\theta_j - \beta} \quad if\ X = 1 \qquad \text{(Equation S2)}$$

$$\frac{P(Y \leq j|X = 0)}{1 - P(Y \leq j|X = 0)} = e^{\theta_j} \quad if\ X = 0 \qquad \text{(Equation S3)}$$

The OR is then

$$\text{OR} = \frac{P(Y \leq j|X = 1)/1 - P(Y \leq j|X = 1)}{P(Y \leq j|X = 0)/1 - P(Y \leq j|X = 0)} = \frac{e^{\theta_j - \beta}}{e^{\theta_j}} = e^{-\beta} \qquad \text{(Equation S4)}$$

Similarly, the OR of the event $Y \geq (j + 1)$ is e^{β}, which is the metric used in our study. An OR associated with category j or above being greater than 1 indicates that the odds of morbidity indicators being in category j or above would increase in individuals with variable $X = 1$ compared to individuals with variable $X = 0$, and vice versa for an OR less than 1.

Note that in this model β is independent of j, meaning that β has the same effect for each of the categories. This is the "proportional odds" assumption. It is possible to test if this assumption is violated, and to use a partial-proportional odds model instead, where the coefficients β are allowed to vary with the category j for the explanatory variable(s) that violate the assumption [5],

$$\text{logit}[P(Y_i \leq j|x_i)] = \log\frac{P(Y_i \leq j|x_i)}{1 - P(Y_i \leq j|x_i)} = \theta_j - x_i\beta + w_i\tilde{\beta}_j - u_i \quad j = 1, \dots, J-1 \qquad \text{(Equation S5)}$$

Here, w_i is a vector of fixed effects covariates for the ith observation and $\tilde{\beta}_j$ is the corresponding set of regression parameters which are allowed to depend on the response category j. In such a case, the OR of being in a category j or above will depend on j [5]. The proportional odds assumption for each explanatory variable was tested using likelihood ratio tests [5]. There was no evidence that the proportional odds assumption was violated for urogenital and intestinal schistosomiasis infection intensities (except for hematuria in children in 2016 but we were not able to fit a partial-proportional odds model) nor for the presence/absence of hybrids. However, the proportional odds assumption was sometimes violated for age, sex, and study site, in which case partial-proportional odds were fitted instead (see Tables S11 and S12).

Table S2. Characteristics of children (*n*=1,319) and adults (*n*=300) in two study sites of Senegal in 2016 and 2017 (complete dataset).

Age group	Year	Site	*Sh* and *Sh-Sb* urogenital infection			*Sm* intestinal infection				Mean age	Proportion of females	Total examined[1]
			Prevalence	Intensity		Prevalence	Intensity					
				Light	Heavy		Light	Moderate	Heavy			
Children	2016	BK	25% (51/204)	19% (38/204)	6% (13/204)	0% (0/206)	-	-	-	9.8	69% (142/205)	*N*=206
		RT	70% (264/378)	43% (162/378)	27% (102/378)	14% (45/318)	6% (20/318)	3% (10/318)	5% (15/318)	10.1	52% (202/391)	*N*=391
	2017	BK	34% (110/328)	21% (69/328)	13% (41/328)	0% (0/204)	-	-	-	10.0	56% (183/328)	*N*=328
		RT	71% (275/387)	58% (223/387)	13% (52/387)	13% (37/290)	7% (21/290)	3% (8/290)	3% (8/290)	10.5	55% (216/392)	*N*=394
Adults	2016	BK	18% (19/104)	14% (15/104)	4% (4/104)	0% (0/108)	-	-	-	40.2	59% (64/108)	*N*=108
		RT	65% (13/20)	60% (12/20)	5% (1/20)	0% (0/20)	-	-	-	36.6	30% (6/20)	*N*=20
	2017	BK	38% (31/81)	36% (29/81)	2% (2/81)	0% (0/58)	-	-	-	33.6	56% (45/81)	*N*=82
		RT	33% (29/87)	31% (27/87)	2% (2/87)	0% (0/40)	-	-	-	33.8	47% (42/90)	*N*=90

BK: Barkedji; RT: Richard Toll; *Sh: Schistosoma haematobium*; *Sh-Sb*: *S. haematobium-S. bovis* hybrids; *Sm*: *S. mansoni*

[1] The total includes individuals that received at least one of the parasitological or clinical examinations, as in Figure 1.

Table S3. Characteristics of children (*n*=378) and adults (*n*=36) with successful follow-up in two study sites of Senegal in 2016 and 2017.

Age group	Year	Site	*Sh* and *Sh-Sb* urogenital infection			*Sm* intestinal infection				Mean age	Proportion of females	Total examined[1]
			Prevalence	Intensity		Prevalence	Intensity					
				Light	Heavy		Light	Moderate	Heavy			
Children	2016	BK	100% (44/44)	73% (32/44)	27% (12/44)	0% (0/44)	-	-	-	9.3	68% (30/44)	*N*=44
	2017	BK	94% (68/72)	58% (42/72)	36% (26/72)	0% (0/39)	-	-	-	9.6	51% (37/72)	*N*=72
		RT	95% (246/259)	76% (196/259)	19% (50/259)	17% (34/196)	9% (18/196)	4% (8/196)	4% (8/196)	10.6	54% (140/261)	*N*=262
Adults	2016	BK	82% (14/17)	65% (11/17)	17% (3/17)	0% (0/17)	-	-	-	34.7	71% (12/17)	*N*=17
	2017	BK	89% (8/9)	89% (8/9)	0% (0/9)	0% (0/7)	-	-	-	28.1	78% (7/9)	*N*=9
		RT	89% (8/9)	89% (8/9)	0% (0/9)	0% (0/7)	-	-	-	29.9	60% (6/10)	*N*=10

BK: Barkedji; RT: Richard Toll; *Sh: Schistosoma haematobium*; *Sh-Sb*: *S. haematobium-S. bovis* hybrids; *Sm*: *S. mansoni*

[1] The total includes individuals with successful follow-up that received at least one of the parasitological or clinical examinations both pre- and post-treatment, as in Figure 1.

Table S4. Characteristics of children (*n*=426) and adults (*n*=46) with genotyped miracidia in two study sites of Senegal in 2016 and 2017.

Age group	Year	Site	Proportion of individuals with *Sh-Sb* miracidia	*Sh* and *Sh-Sb* urogenital infection			*Sm* intestinal infection				Mean age	Proportion of females	Total examined[1]
				Prevalence	Intensity		Prevalence	Intensity					
					Light	Heavy		Light	Moderate	Heavy			
Children	2016	BK	11% (4/35)	100% (35/35)	63% (22/35)	37% (13/35)	0% (0/35)	-	-	-	9.8	66% (23/35)	*N*=35
		RT	63% (106/168)	98% (165/168)	45% (76/168)	53% (89/168)	19% (27/141)	10% (14/141)	3% (5/141)	6% (8/141)	9.9	51% (85/168)	*N*=168
	2017	BK	11% (6/54)	96% (52/54)	46% (25/54)	50% (27/54)	0% (0/33)	-	-	-	9.9	39% (21/54)	*N*=54
		RT	72% (121/169)	92% (155/168)	66% (111/168)	26% (44/168)	17% (21/121)	11% (13/121)	4% (5/121)	2% (3/121)	10.6	54% (90/168)	*N*=169
Adults	2016	BK	23% (3/13)	77% (10/13)	46% (6/13)	31% (4/13)	0% (0/13)	-	-	-	33.8	69% (9/13)	*N*=13
		RT	50% (2/4)	100% (4/4)	100% (4/4)	0% (0/4)	0% (0/4)	-	-	-	35.3	50% (2/4)	*N*=4
	2017	BK	0% (0/16)	81% (13/16)	75% (12/16)	6% (1/16)	0% (0/10)	-	-	-	26.1	50% (8/16)	*N*=16
		RT	100% (13/13)	73% (8/11)	73% (8/11)	0% (0/11)	0% (0/3)	-	-	-	33.7	73% (8/11)	*N*=13

BK: Barkedji; RT: Richard Toll; *Sh: Schistosoma haematobium*; *Sh-Sb*: *S. haematobium-S. bovis* hybrids; *Sm*: *S. mansoni*

[1] The total includes individuals with genotyped miracidia and that received at least one of the parasitological or clinical examinations. Therefore, it is a subset of the complete dataset presented in Table S2.

Table S5. Characteristics of children (*n*=210) and adults (*n*=16) with genotyped miracidia and successful follow-up in two study sites of Senegal in 2016 and 2017.

Age group	Year	Site	Proportion of individuals with *Sh-Sb* miracidia	*Sh* and *Sh-Sb* urogenital infection			*Sm* intestinal infection				Mean age	Proportion of females	Total examined[1]
				Prevalence	Intensity		Prevalence	Intensity					
					Light	Heavy		Light	Moderate	Heavy			
Children	2016	BK	13% (4/31)	100% (31/31)	61% (19/31)	39% (12/31)	0% (0/31)	-	-	-	9.4	68% (21/31)	*N*=31
	2017	BK	15% (5/34)	100% (34/34)	47% (16/34)	53% (18/34)	0% (0/22)	-	-	-	9.9	44% (15/34)	*N*=34
		RT	70% (102/145)	98% (142/145)	69% (100/145)	29% (42/145)	18% (19/104)	10% (11/104)	5% (5/104)	3% (3/104)	10.7	53% (77/144)	*N*=145
Adults	2016	BK	27% (3/11)	82% (9/11)	55% (6/11)	27% (3/11)	0% (0/11)	-	-	-	35.1	73% (8/11)	*N*=11
	2017	BK	0% (0/3)	100% (3/3)	100% (3/3)	0% (0/3)	0% (0/2)	-	-	-	26.3	100% (3/3)	*N*=3
		RT	100% (2/2)	100% (2/2)	100% (2/2)	0% (0/2)	0% (0/1)	-	-	-	37.5	100% (2/2)	*N*=2

BK: Barkedji; RT: Richard Toll; *Sh: Schistosoma haematobium*; *Sh-Sb*: *S. haematobium-S. bovis* hybrids; *Sm*: *S. mansoni*

[1] The total includes individuals with genotyped miracidia and successful follow-up that received at least one of the parasitological or clinical examinations both pre- and post-treatment. Therefore, it is a subset of the dataset presented in Table S3.

Table S6. Prevalence of lower and upper urinary tract lesions assessed by ultrasound among children and adults in two study sites of Senegal in 2017 (number of individuals examined indicated in parentheses).

		Urinary bladder lesions				Dilatation of the ureter		Dilatation of the kidney
		Irregularity	Thickening	Mass	Pseudopolyp	Unilateral	Bilateral	
Children	Barkedji	6% (*n*=303)	12% (*n*=303)	5% (*n*=303)	0% (*n*=303)	2% (*n*=297)	2% (*n*=297)	1% (*n*=297)
	Richard Toll	27% (*n*=335)	26% (*n*=335)	8% (*n*=335)	1% (*n*=335)	6% (*n*=335)	7% (*n*=335)	3% (*n*=335)
Adults	Barkedji	16% (*n*=19)	5% (*n*=19)	11% (*n*=19)	0% (*n*=19)	0% (*n*=19)	0% (*n*=19)	0% (*n*=19)
	Richard Toll	24% (*n*=41)	17% (*n*=41)	0% (*n*=41)	0% (*n*=41)	0% (*n*=41)	2% (*n*=41)	0% (*n*=41)

Figure S2. Example of ultrasound images of bladder lesions (credit: C. B. Fall).

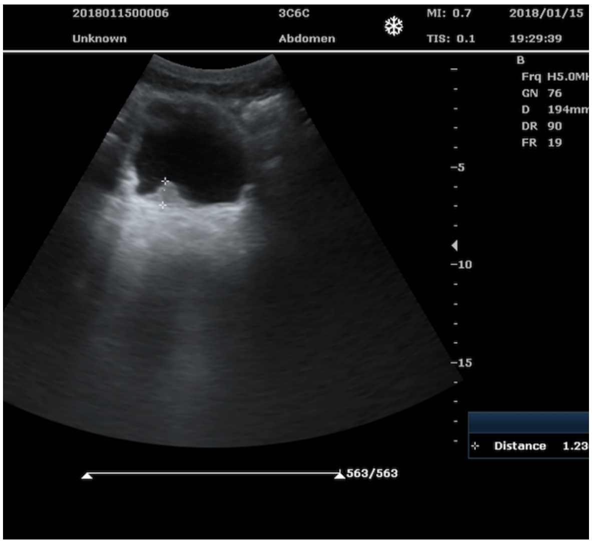

Mass of the bladder wall

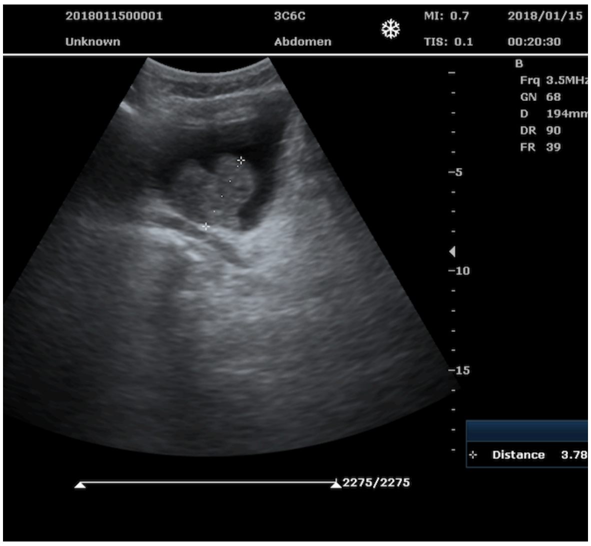

Mass of the bladder wall

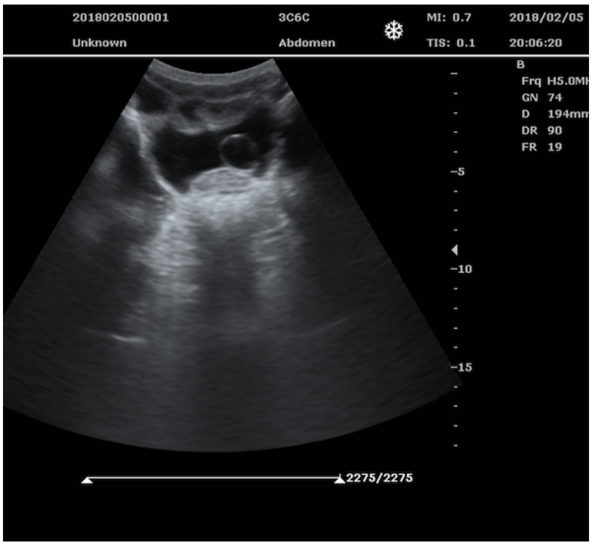

Ureterocele

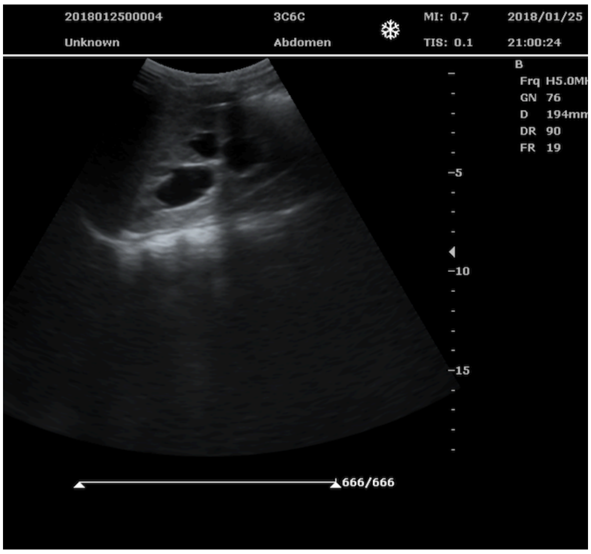

Dilatation of the kidney

Table S7. Odds ratios [95% confidence intervals] and *p*-values (in parentheses) of anemia in 2016 and 2017 and hepatomegaly being in category *j* or above among children in Senegal, depending on schistosomiasis infection intensity and adjusted for age, sex, and study site. Only individuals with complete data on anemia or hepatomegaly, urogenital or intestinal infection intensities, age, sex, and study site were included in the cumulative link logistic regression models (for anemia *n*=453 in 2016 and *n*=269 in 2017; for hepatomegaly *n*=137).

		**ANEMIA 2016 (*j* = mild, moderate, or severe)**		
		***Sh* and *Sh-Sb* urogenital infection intensity**		
		None	Light	Heavy
**_Sm_ intestinal infection intensity**	None	1	1.15 [0.89 – 1.49] (*p*=0.29)	1.32 [0.79 – 2.21] (*p*=0.29)
	Light	1.47 [0.82 – 2.64] (*p*=0.20)	1.37 [0.93 – 2.02] (*p*=0.11)	1.28 [0.67 – 2.43] (*p*=0.45)
	Moderate	2.16 [0.67 – 6.96] (*p*=0.20)	1.64 [0.87 – 3.08] (*p*=0.12)	1.24 [0.43 – 3.57] (*p*=0.69)
	Heavy	3.18 [0.55 – 18.4] (*p*=0.20)	1.96 [0.79 – 4.83] (*p*=0.15)	1.20 [0.26 – 5.61] (*p*=0.81)
		**ANEMIA 2017 (*j* = mild, moderate, or severe)**		
		***Sh* and *Sh-Sb* urogenital infection intensity**		
		None	Light	Heavy
**_Sm_ intestinal infection intensity**	None	1	0.80 [0.52 – 1.23] (*p*=0.31)	0.64 [0.27 – 1.51] (*p*=0.31)
	Light	0.60 [0.20 – 1.75] (*p*=0.35)	0.87 [0.48 – 1.57] (*p*=0.64)	1.27 [0.47 – 3.39] (*p*=0.64)
	Moderate	0.35 [0.04 – 3.05] (*p*=0.35)	0.94 [0.36 – 2.46] (*p*=0.90)	2.49 [0.50 – 12.3] (*p*=0.26)
	Heavy	0.21 [0.01 – 5.34] (*p*=0.35)	1.02 [0.26 – 4.04] (*p*=0.98)	4.91 [0.47 – 51.2] (*p*=0.18)
		**HEPATOMEGALY (*j* = moderate or severe)**		
		***Sh* and *Sh-Sb* urogenital infection intensity**		
		None	Light	Heavy
**_Sm_ intestinal infection intensity**	None	1	0.93 [0.52 – 1.66] (*p*=0.81)	0.87 [0.28 – 2.74] (*p*=0.81)
	Light	0.26 [0.02 – 4.50] (*p*=0.36)	0.53 [0.17 – 1.65] (*p*=0.27)	1.07 [0.07 – 17.5] (*p*=0.96)
	Heavy	0.02 [0.000004 – 91.0] (*p*=0.36)	0.17 [0.008 – 3.69] (*p*=0.26)	1.61 [0.0004 – 5,989] (*p*=0.91)

Sh: Schistosoma haematobium; *Sh-Sb*: *S. haematobium-S. bovis* hybrids; *Sm*: *S. mansoni*

Table S8. Odds ratio [95% confidence intervals] and *p*-values (in parentheses) of various morbidity indicators being in category *j* or above among adults in Senegal in 2016 and 2017, depending on urogenital schistosomiasis infection intensity and adjusted for age, sex, and study site.

Morbidity indicator	*n*[1]	***Sh* and *Sh-Sb* urogenital infection intensity**	
		Light	Heavy
Hematuria 2016 (*j* = +, ++ or +++)	122	7.60 [3.42 – 18.4] (*p*<0.0001)	57.7 [11.8 – 340] (*p*<0.0001)
Hematuria 2017 (*j* = +, ++ or +++)	157	4.22 [2.21 – 8.06] (*p*<0.0001)	17.8 [4.90 – 65.0] (*p*<0.0001)
Anemia 2016 (*j* = mild, moderate, or severe)	42	1.21 [0.40 – 3.51] (*p*=0.72)	1.48 [0.16 – 12.3] (*p*=0.72)
Anemia 2017 (*j* = mild, moderate, or severe)	41	1.89 [0.39 – 9.46] (*p*=0.43)	3.57 [0.15 – 89.6] (*p*=0.43)
Global score[2] (*j* = positive)	58	4.48 [1.52 – 14.6] (*p*=0.0085)	20.1 [2.30 – 213] (*p*=0.0085)
Bladder wall calcification (*j* = present)	39	17.0 [2.70 – 208] (*p*=0.0084)	288 [7.31 – 43,451] (*p*=0.0084)

Sh: Schistosoma haematobium; *Sh-Sb*: *S. haematobium-S. bovis* hybrids

[1] Only individuals with complete data on each morbidity indicator, urogenital or intestinal infection intensities, age, sex, and study site were included in the cumulative link logistic regression models.

[2] The global score is the sum of the bladder and the upper tract intermediate scores.

Table S9. Odds ratio [95% confidence intervals] and *p*-values (in parentheses) of urinary bladder lesions as assessed by ultrasound among children in Senegal in 2017, depending on schistosomiasis infection intensity and adjusted for age, sex, and study site. Only individuals with complete data on urinary bladder lesions, urogenital or intestinal infection intensities, age, sex, and study site were included in the cumulative link logistic regression models (*n*=424).

		**IRREGULARITY**		
		***Sh* and *Sh-Sb* urogenital infection intensity**		
		None	Light	Heavy
**_Sm_ intestinal infection intensity**	None	1	2.20 [1.39 – 3.48] (*p*=0.0074)	4.84 [1.94 – 12.1] (*p*=0.0074)
	Light	2.44 [0.60 – 9.92] (*p*=0.21)	1.93 [0.98 – 3.79] (*p*=0.060)	1.53 [0.35 – 6.65] (*p*=0.57)
	Moderate	5.96 [0.36 – 98.5] (*p*=0.21)	1.70 [0.55 – 5.18] (*p*=0.35)	0.48 [0.03 – 7.12] (*p*=0.60)
	Heavy	14.5 [0.22 – 977] (*p*=0.21)	1.49 [0.30 – 7.44] (*p*=0.63)	0.15 [0.003 – 8.41] (*p*=0.36)
		**THICKENING**		
		***Sh* and *Sh-Sb* urogenital infection intensity**		
		None	Light	Heavy
**_Sm_ intestinal infection intensity**	None	1	2.62 [1.72 – 4.01] (*p*<0.0001)	6.88 [2.95 – 16.1] (*p*<0.0001)
	Light	1.00 [0.28 – 3.55] (*p*=0.99)	2.84 [1.39 – 5.81] (*p*=0.0042)	8.09 [2.78 – 23.6] (*p*=0.00012)
	Moderate	1.00 [0.08 – 12.6] (*p*=0.99)	3.08 [0.95 – 10.0] (*p*=0.061)	9.52 [1.62 – 55.9] (*p*=0.013)
	Heavy	1.00 [0.02 – 44.7] (*p*=0.99)	3.34 [0.63 – 17.8] (*p*=0.16)	11.2 [0.84 – 149] (*p*=0.067)
		**MASS**		
		***Sh* and *Sh-Sb* urogenital infection intensity**		
		None	Light	Heavy
**_Sm_ intestinal infection intensity**	None	1	5.54 [2.56 – 12.0] (*p*<0.0001)	30.7 [6.54 – 144] (*p*<0.0001)
	Light	2.18 [0.45 – 10.5] (*p*=0.33)	10.6 [3.52 – 32.0] (*p*<0.0001)	51.6 [10.2 – 262] (*p*<0.0001)
	Moderate	4.75 [0.21 – 110] (*p*=0.33)	20.3 [3.89 – 106] (*p*=0.00035)	86.8 [8.94 – 844] (*p*=0.00012)
	Heavy	10.4 [0.09 – 1150] (*p*=0.33)	38.9 [4.05 – 374] (*p*=0.0015)	146 [6.20 – 3,441] (*p*=0.0020)

Sh: Schistosoma haematobium; Sh-Sb: *S. haematobium-S. bovis* hybrids; *Sm*: *S. mansoni*

Table S10. Odds ratio [95% confidence intervals] and *p*-values (in parentheses) of upper urinary tract lesions being in category *j* or above as assessed by ultrasound among children in Senegal in 2017, depending on schistosomiasis infection intensity and adjusted for age, sex, and study site. Only individuals with complete data on upper urinary tract lesions, urogenital or intestinal infection intensities, age, sex, and study site were included in the cumulative link logistic regression models (*n*=419).

		**DILATATION OF THE URETER (*j* = unilateral or bilateral)**		
		***Sh* and *Sh-Sb* urogenital infection intensity**		
		None	Light	Heavy
**Sm intestinal infection intensity**	None	1	2.12 [1.23 – 3.64] (*p*=0.0068)	4.47 [1.51 – 13.2] (*p*=0.0068)
	Light	0.80 [0.15 – 4.11] (*p*=0.79)	1.81 [0.76 – 4.32] (*p*=0.18)	4.10 [1.07 – 15.7] (*p*=0.040)
	Moderate	0.64 [0.02 – 16.9] (*p*=0.79)	1.54 [0.35 – 6.70] (*p*=0.56)	3.75 [0.40 – 35.4] (*p*=0.25)
	Heavy	0.51 [0.004 – 69.5] (*p*=0.79)	1.32 [0.16 – 10.9] (*p*=0.80)	3.43 [0.13 – 92.6] (*p*=0.46)
		**DILATATION OF THE KIDNEY (*j* = present)**		
		***Sh* and *Sh-Sb* urogenital infection intensity**		
		None	Light	Heavy
**Sm intestinal infection intensity**	None	1	0.95 [0.34 – 2.67] (*p*=0.92)	0.90 [0.11 – 7.14] (*p*=0.92)
	Light	1.61 [0.14 – 19.0] (*p*=0.71)	1.14 [0.28 – 4.67] (*p*=0.86)	0.81 [0.05 – 14.4] (*p*=0.89)
	Moderate	2.58 [0.02 – 360] (*p*=0.71)	1.37 [0.15 – 12.6] (*p*=0.78)	0.73 [0.005 – 115] (*p*=0.90)
	Heavy	4.15 [0.003 – 6839] (*p*=0.71)	1.65 [0.07 – 38.1] (*p*=0.75)	0.66 [0.0004 – 1,186] (*p*=0.91)

Sh: Schistosoma haematobium; *Sh-Sb*: *S. haematobium-S. bovis* hybrids; *Sm*: *S. mansoni*

Table S11. Odds ratio [95% confidence intervals] and *p*-values (in parentheses) of various morbidity indicators being in category *j* or above among children in Senegal in 2016 and 2017, depending on age, sex, and study site.

Morbidity indicator	*n*[1]	*j*	Study site (OR RT *vs* BK)	Sex (OR Boys *vs* Girls)	Age (OR per year)
Hematuria 2016	509	+	1.17 [0.52 – 2.60] (*p*=0.70)	1.19 [0.75 – 1.88] (*p*=0.47)	0.92 [0.84– 1.01] (*p*=0.076)
		++		1.54 [0.93 – 2.55] (*p*=0.092)	
		+++		1.03 [0.61 – 1.74] (*p*=0.92)	
Hematuria 2017	472	+	7.27 [3.08 – 17.1] (*p*<0.0001)	1.37 [0.90 – 2.09] (*p*=0.15)	1.02 [0.92 – 1.13] (*p*=0.73)
		++	3.59 [1.52 – 8.49] (*p*=0.0036)		
		+++	3.17 [1.31 – 7.65] (*p*=0.010)		
Anemia 2016	453	mild	1.07 [0.69 – 1.66] (*p*=0.76)	1.49 [1.03 – 2.16] (*p*=0.036)	0.99 [0.91 – 1.07] (*p*=0.81)
		moderate	1.74 [1.08 – 2.80] (*p*=0.024)		0.93 [0.85 – 1.01] (*p*=0.071)
		severe	10.3 [2.40 – 44.1] (*p*=0.0017)		0.63 [0.52 – 0.76] (*p*<0.0001)
Anemia 2017	269	mild, moderate or severe	-	1.87 [1.16 – 3.03] (*p*=0.010)	1.01 [0.89 – 1.16] (*p*=0.83)
Global score[2]	419	positive	5.10 [2.21 – 11.8] (*p*=0.00014)	3.59 [2.03 – 6.37] (*p*<0.0001)	0.92 [0.80 – 1.06] (*p*=0.27)
Bladder intermediate score	424	likely or very likely	2.73 [1.32 – 5.62] (*p*=0.0068)	2.32 [1.43 – 3.77] (*p*=0.0065)	0.91 [0.80 – 1.03] (*p*=0.12)
Upper tract intermediate score	419	positive	3.30 [1.43 – 7.61] (*p*=0.0050)	2.43 [1.24 – 4.80] (*p*=0.010)	1.00 [0.85 – 1.18] (*p*=0.96)
Bladder wall calcification	419	present	7.04 [2.89 – 17.2] (*p*<0.0001)	2.19 [1.18 – 4.04] (*p*=0.012)	1.08 [0.93 – 1.25] (*p*=0.31)
Hepatomegaly	137	Moderate or severe	6.35 [2.30 – 17.5] (*p*=0.00036)	1.93 [0.91 – 4.09] (*p*=0.088)	0.66 [0.54 – 0.81] (*p*<0.0001)

[1] Only individuals with complete data on each morbidity indicator, urogenital or intestinal infection intensities, age, sex, and study site were included in the cumulative link logistic regression models.
[2] The global score is the sum of the bladder and the upper tract intermediate scores.

Table S12. Odds ratio [95% CI] and *p*-values (in parentheses) of various morbidity indicators being in category *j* or above in adults of Senegal in 2016 and 2017, depending on age, sex, and study site.

Morbidity indicator	*n*[1]	*j*	Study site (OR RT *vs* BK)	Sex (OR Men *vs* Women)	Age (OR per year)
Hematuria 2016	122	+, ++ or +++	2.36 [0.83 – 6.68] (*p*=0.10)	0.44 [0.20 – 0.94] (*p*=0.037)	1.02 [0.99 – 1.04] (*p*=0.29)
Hematuria 2017	157	+	1.27 [0.65 – 2.49] (*p*=0.49)	0.55 [0.28 – 1.08] (*p*=0.081)	1.02 [0.99 – 1.05] (*p*=0.21)
		++			0.99 [0.96 – 1.03] (*p*=0.59)
		+++			0.97 [0.93 – 1.01] (*p*=0.13)
Anemia 2016	42	mild, moderate or severe	-	0.21 [0.03 – 0.98] (*p*=0.071)	0.99 [0.93 – 1.05] (*p*=0.70)
Anemia 2017	41	mild, moderate or severe	-	0.96 [0.26 – 3.62] (*p*=0.95)	1.00 [0.94 – 1.07] (*p*=0.89)
Global score[2]	58	positive	1.27 [0.33 – 5.24] (*p*=0.73)	1.32 [0.39 – 4.59] (*p*=0.66)	0.95 [0.90 – 1.01] (*p*=0.10)
Bladder wall calcification	39	present	-	5.13 [0.63 – 75.3] (*p*=0.17)	0.96 [0.87 – 1.05] (*p*=0.42)

[1] Only individuals with complete data on each morbidity indicator, urogenital or intestinal infection intensities, age, sex, and study site were included in the cumulative link logistic regression models.
[2] The global score is the sum of the bladder and the upper tract intermediate scores.

REFERENCES

1. WHO Haemoglobin Concentrations for the Diagnosis of Anaemia and Assessment of Severity Available online: http://www.who.int/vmnis/indicators/haemoglobin/en/ (accessed on 6 February 2020).
2. WHO *Ultrasound in Schistosomiasis: A Practical Guide to the Standard Use of Ultrasonography for Assessment of Schistosomiasis-Related Morbidity*; World Health Organization, UNDP/World Bank/WHO Special Programme for Research and Training in Tropical Diseases: Niamey, Niger, 1996; p. 55;.
3. Richter, J.; Domingues, A.L.D.; Barata, C.H.; Prata, A.R.; Lambertucci, J.R. Report of the Second Satellite Symposium on Ultrasound in Schistosomiasis. *Mem. Inst. Oswaldo Cruz* **2001**, *96*, 151–156, doi:10.1590/S0074-02762001000900023.
4. Christensen, R.H.B. *Ordinal - Regression Models for Ordinal Data. R Package Version 2019.12-10*; 2019;
5. Christensen, R.H.B. Cumulative Link Models for Ordinal Regression with the R Package Ordinal Available online: https://cran.r-project.org/web/packages/ordinal/vignettes/clm_article.pdf (accessed on 3 June 2020).
